# T-Cell Compartmentalization and Functional Adaptation in Autoimmune Inflammation: Lessons From Pediatric Rheumatic Diseases

**DOI:** 10.3389/fimmu.2019.00940

**Published:** 2019-05-09

**Authors:** Gerdien Mijnheer, Femke van Wijk

**Affiliations:** Laboratory of Translational Immunology, Wilhelmina Children's Hospital, University Medical Center Utrecht, Utrecht University, Utrecht, Netherlands

**Keywords:** T-cell adaptation, autoimmune-inflammation, regulatory T-cell, CD8 T-cell, tissue-resident memory T-cell, clonal expansion, JIA, SLE

## Abstract

Chronic inflammatory diseases are characterized by a disturbed immune balance leading to recurring episodes of inflammation in specific target tissues, such as the joints in juvenile idiopathic arthritis. The tissue becomes infiltrated by multiple types of immune cell, including high numbers of CD4 and CD8 T-cells, which are mostly effector memory cells. Locally, these T-cells display an environment-adapted phenotype, induced by inflammation- and tissue-specific instructions. Some of the infiltrated T-cells may become tissue resident and play a role in relapses of inflammation. Adaptation to the environment may lead to functional (re)programming of cells and altered cellular interactions and responses. For example, specifically at the site of inflammation both CD4 and CD8 T-cells can become resistant to regulatory T-cell-mediated regulation. In addition, CD8 and CD4 T-cells show a unique profile with pro- and anti-inflammatory features coexisting in the same compartment. Also regulatory T-cells are neither homogeneous nor static in nature and show features of functional differentiation, and plasticity in inflammatory environments. Here we will discuss the recent insights in T-cell functional specialization, regulation, and clonal expansion in local (tissue) inflammation.

## Introduction

A typical hallmark of immune-mediated inflammatory diseases (IMIDs) is the intermittent presence of inflammation that manifest in specific target tissues. In affected tissues, activated antigen-specific T-cells with a memory phenotype are present. The combination of memory T-cell infiltrated at affected sites and genetic associations have pointed to T-cells as key players in the pathophysiology of chronic inflammatory diseases. Due to the ongoing inflammation in target tissues there is an increasing risk for collateral tissue damage. Especially in children this can have long-term effects and consequences. Understanding the biology of immune cells that are actively involved in the local inflammatory process is crucial for the development of therapeutic approaches. However, because of technical challenges, the vast majority of data in human studies comes from peripheral blood cells. With the emergence of novel techniques including (single-cell) RNA-sequencing and mass cytometry it is now possible to unravel T-cell signatures in a local (tissue) setting. Here, we will discuss the most recent findings on human T-cell functional programming and adaptation at the site of chronic inflammation with a focus on pediatric rheumatologic disease.

## T-cells at the Site of Inflammation

Inflammation is a process aimed at eliminating the triggering agent, but in chronic inflammation the (auto)antigens persist, inducing a sustained inflammation. When inflammation does resolve, a population of antigen-specific T-cells may remain in the tissue as memory cells and become tissue resident (1), the so called tissue-resident memory T-cells (Trm). This makes them unique compared to central memory, effector memory and naïve T-cells that recirculate within lymphoid organs and blood and/or lymphatics. A hallmark of Trm cells is their ability to respond quickly and robustly after re-encountering the antigen as well as the expression of inhibitory molecules to keep them in check. In steady-state, Trm play an important role in tissue homeostasis and protection. After immune re-activation of Trm, circulating immune cells are attracted to the site of inflammation as well, resulting in local accumulation of antigen-specific memory T-cells. So both Trm and infiltrating T-cells that might eventually become Trm themselves, may actively participate during the inflammatory response. Because of their vigilance and specific localization in tissues, Trm are suspected to play a dominant role in the typical relapsing remitting course of chronic inflammatory diseases. Not much is known about the dynamics and interaction between both memory subsets. An accumulation of both CD4^+^ and CD8^+^ T-cells are found at sites of chronic inflammation. However, research on the involvement of T-cells in disease pathogenesis has mainly focused on CD4^+^ T-cells, whereas CD8^+^ T-cells have been the focus of the Trm research field. In this review we summarize the findings on CD8^+^, CD4^+^, and FOXP3^+^ regulatory T-cell (Treg) subsets in the contexts of local (tissue) adaptation and functional differentiation. In line with the divarication of T helper cells, with each subset having a specified function in the immune system, it is now becoming apparent that specialization is also true for T-cells present in different tissues and types of inflammation. The local acquisition of additional or adjusted functions and phenotypes will be referred to as adaptation.

### CD4^+^ T Helper Responses in Local Pathogenesis

For a long time, CD4^+^ T have been recognized as central players in the immune-pathogenesis of autoimmune diseases, which is supported by strong associations of rheumatic diseases with MHC class II alleles (2). The CD4^+^ T-cell population is comprised of several T helper subsets that develop after the T-cell receptor (TCR) on naïve CD4^+^ T-cells interacts with activated antigen presenting cells (APCs). Next to this TCR stimulation, co-stimulation, subsequent signaling and cytokines in the micro-environment are important in determining the fate of a specific subset, by activating signaling molecules that establish a lineage-specific enhancer landscape and lead to the expression of master transcription factors. Those, together with a complex network of accessory transcription factors, can coordinate cellular differentiation programs committed toward a lineage, while simultaneously repressing the developmental programs of opposing Th lineages (3). For many years the only identified lineages were Th1 and Th2 subsets, with IFNγ producing Th1 cells being specialized in cell-mediated immune responses against intracellular bacteria and Th2 cells producing IL-4 and IL-13 targeting helminths. The discovery of other subsets, such as Th17, Th9, and T follicular helper (Tfh) cells has shifted the paradigm of two opposing lineages (4–6). Th17 cells, producing various cytokines including IL-17, IL-22, and GM-CSF, induce defense against fungi and extracellular bacteria and are crucial for the maintenance of mucosal homeostasis (7). Despite their relatively recent discovery, Th17 cells are implicated in the pathogenesis of many human (autoimmune-) diseases. This also accounts for Tfh cells that provide help to cognate B cells to produce high affinity antibodies and memory B cells and as such control humoral immunity (8). Recent reports indicate that Th9 cells, mainly producing IL-9, may also be involved in the pathogenesis of auto-immune diseases, possibly by promoting Th17 differentiation (4).

Many immune-mediated diseases are associated with aberrant Th responses. In the lamina propria of Crohn's disease, the synovial fluid (SF) of Juvenile Idiopathic Arthritis (JIA) and Rheumatoid Arthritis (RA) patients and renal tissue of Systemic Lupus Erythematosus (SLE) patients for example, Th1 cells are implicated in disease pathogenesis (9–12). In JIA, a mixed Th17/Th1 phenotype is also found in inflamed joints, capable of producing both IL-17 and IFNγ. The presence of this subset correlates with disease activity (13, 14) and its IL-17-producing capacity is associated with CD161 expression (15). Interestingly, this subset seems to be present specifically in inflammatory environments, and can be a transiently induced from Th17 cells upon exposure to IL-12 and/or TNFα (13, 14, 16). Whereas, the Th1 lineage is shown to be fairly stable, the Th17 lineage is known for its instability and is severely impacted by the environment (17). In line with this, the mixed Th1/Th17 phenotype likely derives from Th17 cells instead of Th1 cells (18). Next to pathogenic Th17 cells, non-pathogenic Th17 cells have been described in autoimmune diseases as well [reviewed in (19)]. A recent paper found delayed IL-10 production in about 25% of activated human Th17 clones in culture (20). This indicates that IL-10 production is an intrinsic property of a subset of Th17 cells after antigenic stimulation, perhaps to regulate and balance the immune response. Transcriptional analysis of the IL-10^+^ and IL-10^−^ Th17 clones demonstrated immune-regulatory and tissue-resident properties of IL-10 producing Th17 cells, and a pro-inflammatory profile of IL-10^−^ Th17 cells with high CCR7 expression, which may indicate circulatory properties (20). Local pathogenic Th17 cells have been described in multiple autoimmune disease [reviewed in (17)]. In muscle of JDM patients and affected kidneys of SLE patients, IL-17 producing cells are increased (21–23). One mechanism that explains the elevated production of IL-17 in JIA and SLE is the increased expression of the transcription factor cAMP-responsive element modulator (CREM)α. This induces repression of IL-2 transcription but also epigenetic changes of the IL-17A locus, resulting in enhanced IL-17A promoter activity (24, 25). Although evidence for involvement of the IL-17 signaling pathway in SLE pathogenesis is expanding, direct interference with IL-17 or it's receptor does not seem to be effective, at least in mouse models (26, 27). Also in JIA, IL-17 blockade is not part of standard treatment. This might be related to the pathogenic role of another CD4^+^ subset, Tfh cells that is increased in inflamed tissues of RA and SLE patients and located near B cells in affected kidneys in SLE patients (28, 29). Interestingly, STAT-3 and IL-21, signature molecules shared by Th17 and Tfh cells, are heavily implicated in SLE pathology and are capable of inducing autoreactive B cells (30, 31). Alternatively, the lack of IL-17 blockade efficacy in JIA might be explained by Th17 cells that can be polarized in the inflamed joints to shift toward the so called non-classic Th1 subset. These cells have been shown to lose the ability to produce IL-17 while maintaining both RORc and CD161 expression and produce high levels of IFNγ, in line with the mixed Th1/Th17 cells described above (18). Altogether, overactive CD4^+^ T-cells are present at the site of human inflammation and represent a mixed population of which especially Th17 cells show plasticity.

#### A Continuum of Th Cell Fates

The presence of mixed CD4^+^ phenotypes found in human sites of autoimmune inflammation is of great interest, but might not be inflammation-specific. Recent studies using novel technologies have revealed a continuum of cell fates rather than limited and distinct Th subsets in healthy tissues. Mass cytometry measuring T-cell trafficking receptor and cytokine expression in eight different human tissues revealed tissue-specific and unique T-cell phenotypes (32). This indicates that T-cells cannot be easily classified into separate lineages across human tissues. Furthermore, multi-color cytometry of peripheral blood cells of a healthy human cohort showed substantial subject-to-subject differences in T-cell populations that yet remained relatively stable for months within individuals (33). Age and disease associated genetic polymorphisms were identified as important factors in the identified variation. These publications highlight the importance of age and tissue influences on T cells [reviewed in (34)]. On top of this homeostatic variety, inflammation will probably add another layer of complexity by driving local tissue cells into an adapted phenotype.

### CD8^+^ T Helper Responses in Local Pathogenesis

Although long neglected in autoimmune diseases compared to CD4^+^ T-cells, CD8^+^ T-cells are equipped with different capacities by which they can contribute to the inflammatory process. For example, CD8^+^ T-cells have cytolytic activity, produce pro-inflammatory cytokines and can react to self-antigens upon cross-presentation (35). In several chronic inflammatory diseases CD8^+^ T-cells are described to be present in the inflamed tissues and increased CD8^+^ T-cell numbers are associated poor prognosis in several rheumatic diseases including JIA, RA and SLE (36–39). CD8^+^ T-cells form a diverse group of cells but in contrast to CD4^+^ T-cells subsets are less well-defined. The phenotype of CD8^+^ T-cells at the inflamed site of human autoimmune arthritis is heterogeneous, with both pro- and anti-inflammatory features [reviewed in (35)]. In SF of RA patients for example, CD8^+^ T-cells are characterized by increased expression of activation markers (CD80, CD86, CD25), pro-inflammatory cytokines like IL-6 and TNFα, and with a proliferative signature, but also by elevated levels of negative co-stimulatory markers, such as TIM-3 and PD-1 (36, 40, 41). In affected kidneys of SLE patients, the majority of CD8^+^ T-cells are located in the periglomerular regions where tissue damage occurs, and this infiltrate is correlated with renal injury (42). Despite multiple studies reporting CD8^+^ T-cells accumulation in SLE affected tissues, the phenotype of local CD8^+^ T-cells remains largely unexplored. One study reports a differentiated effector memory phenotype with loss of CD28 on infiltrating CD8^+^ T-cells, indicating active involvement of these cells in disease pathology (43). However, although counter-intuitive for effector cells in an autoimmune environment, a recent study shows that kidney-infiltrating T-cells are metabolically and functionally “exhausted” in three mouse models of lupus nephritis (44). The term “exhausted” stems from numerous studies on CD8^+^ T-cells in chronic viral infections and, to a lesser extent, in cancers. Chronic antigen exposure in these settings goes along with the upregulation of negative co-stimulatory markers, such as PD-1 in combination with reduced secretion of effector cytokines and proliferation. This has led to the hypothesis that these cells are terminally differentiated and severely functionally impaired (45, 46). However, recently this concept was challenged by the discovery that PD-1^+^ CD8^+^ T-cells are functionally adapted cells able to control the viral load or tumor cells without causing excessive immune pathology, and can be therapeutically reinvigorated by blocking PD-1/PD-L1 interaction (47, 48).

#### Chronic Stimulation in Auto-Immune Inflammation: From an Exhausted to a Trm Phenotype

Not much is known about the functional profile of tissue-specific memory CD8^+^ T-cell in human chronic autoimmune diseases. Like CD8^+^ T-cells in infectious diseases and tumors, these cells are chronically activated. However, instead of resulting in strengthened regulation as is described for the aforementioned conditions, chronic stimulation in autoimmune diseases seems to lead toward overzealous and pathogenic effector functions. Previous observations from peripheral blood derived CD8^+^ T-cells show that the transcriptional signature reflecting exhaustion is associated with poor clearance of chronic viral infection, but conversely predicts better prognosis in multiple auto-immune diseases, including SLE (49). A recent paper addressing the enriched PD-1^+^ CD8^+^ T-cell population in SF of JIA patients is the first to study this cell subset locally, derived from the site of inflammation in humans (50). In this setting, PD-1 expressing CD8^+^ T-cells maintain their effector function, such as pro-inflammatory cytokine production, cytotoxic profile, and use of glycolysis as a metabolic pathway and thereby are suspected to have a detrimental role in autoimmune tissue damage. Strong inflammatory signals, and high levels of soluble PD-1 that block interaction with APCs (51), may overrule or hamper PD-1-signaling in CD8^+^ T-cells in SF of JIA patients. In line with this, Odorizzi et al. have shown that PD-1 expression is not a prerequisite for exhaustion to occur, by using a mouse model of chronic viral infection with genetic absence of PD-1. In this model, PD-1 prevented CD8^+^ T-cells overstimulation and apoptosis, as the absence of PD-1 led to more cytotoxic but terminally differentiated CD8^+^ T-cells (47). It is tempting to speculate that this mechanism may also play a role in local auto-immune inflammation, leading to survival of auto-antigen induced clonally expanding effector cells.

In line with this, other T-cell subsets have been described that may be induced by chronic stimulation and acquire a pathogenic role. The latter is reflected by the secretion of inflammatory cytokines and expression of granzymes and perforin. These include CD8^+^ T-cells expressing CD57 (52, 53) and CD4^+^/CD8^+^ T-cells that have downregulated CD28, so called CD28^null^ cells (54, 55). Some reports have demonstrated that these cells are also present in SF of RA patients, whereas others failed to confirm this (56, 57). There are indications that these chronically activated CD4^+^ and CD8^+^ T-cells cells are induced by CMV infection in RA patients (58), although a causative linkage has not been established thus far (54, 59).

Interestingly, the combined cytotoxic and regulatory profile of CD8^+^ T-cells, defined by increased expression of effector molecules, such as Granzyme B on the one hand, and of negative co-stimulatory molecules on the other hand, is also typical for Trm cells (60). In line with this, the PD-1^+^ CD8^+^ cells in SF were shown to be enriched for a Trm transcriptional profile compared to the PD-1^−^ CD8^+^ T-cells from the same environment (50). Furthermore, Trm are defined by CD69 expression and downregulation of S1PR1, which is also found on PD-1^+^ CD8^+^ cells derived from SF of JIA patients. So local CD8^+^ T-cells from the site of autoimmune inflammation in JIA cannot be classified as exhausted, despite some overlapping features, but share much of their profile with Trm cells. Interestingly, tumor-infiltrating CD8+ T-cells in human cancers also display a Trm profile (61). Moreover, a recent paper describing the transcriptional, metabolic, and functional signatures of intra-tumoral PD-1^+^ CD8^+^ T-cells has revealed that, next to many commonalities, such as impaired cytokine production, these cells also differ from exhausted cells as described in chronic infections (62). The intra-tumoral CD8^+^ T-cells showed increased proliferation and glycolysis, and lack of enrichment of the exhausted T-cell gene signature (62), as was observed in SF of JIA patients. The interpretation now arises that CD8^+^ “exhausted” cells are a heterogeneous group of memory cells with diverse differentiation states but all driven by persistence of antigen that induces upregulation of inhibitory receptors, and with functional properties that are influenced by the environment (63, 64). This concept shares many features with the current view on Trm cells as they are also regarded as highly specialized cells with a tissue adapted profile, tightly regulated to prevent excessive tissue damage. The commonalities between CD8^+^ T-cells at inflammatory sites and the Trm profile suggest interplay between inflammation and tissue residency. Indeed, several studies indicate that inflammation is the trigger for initial homing of Trm cells, by providing the migratory signals needed to direct them to tissues (61). PD-1 expressing CD8^+^T-cells in an inflammatory exudate, such as SF of JIA could be tissue derived, but how they developmentally relate to Trm cells remains unknown. All in all, local CD8^+^ T-cells situated in the affected tissue of chronic inflammation are heterogeneous with both effector and regulatory responses that are highly influenced by chronic inflammation, possibly with disease specific profiles.

### FOXP3 Regulatory T-Cells Responses in Local Pathogenesis

Overzealous CD4^+^ Th responses carry the risk of initiating detrimental pro-inflammatory responses that can result in collateral tissue damage. Thus, the maintenance of immune homeostasis and prevention of immunopathology requires tight regulatory mechanisms. Regulatory T-cells (Treg) are a subset of CD4^+^ T-cells with unique homeostatic functions. Absence of the Treg master transcription FOXP3 leads to fatal multi-organ autoimmunity in mice and men (65, 66). The capacity of Treg to dampen immune responses have made them attractive therapeutic targets in diverse settings, such as in autoimmune diseases, transplantation, and cancer. The best discriminative surface markers for Treg are high expression of CD25 (IL-2 receptor α) in combination with low expression of CD127 (IL-7 receptor α). Early studies often used solely high CD25 expression to identify or purify Treg, resulting in contamination with activated conventional CD4+ T-cells, and contradicting data.

#### Effector and Polarized Treg

Traditionally, the Treg lineage was considered as a homogeneous group of committed cells with suppressive capacities toward other immune cells. As it increasingly becoming apparent that most, if not all, immune cells have the capacity to adapt to their environment, data from the last decade demonstrated that Treg are perhaps the most heterogeneous in phenotype and function (67). Their high turnover and sensitivity to environmental signals leads to a large degree of adaptation that allows Treg to control diverse immune responses and even to exert tissue-specific functions. In that respect novel Treg phenotypes have been identified that differ markedly from their naïve recirculating counterparts (central Treg). Liston and Gray have proposed the model of environment-instructed effector Treg and polarized Treg differentiation (68). Effector Treg, or eTreg, are characterized in humans by Foxp3^high^CD25^high^CD45RA^low^ expression representing a small fraction of circulating Treg (69). They have signs of recent antigen encounter, have an heightened activation status and migratory potential, and express markers similar to activated conventional CD4^+^ T-cells while maintaining Treg functions (68). For example, increased expression of Foxp3, as well as typical functional markers, such as ICOS and CTLA4 is observed in eTreg (69, 70). Polarized (tissue-resident) Treg are present in non-lymphoid tissues, express specific homing receptors and exert tissue-specific functions and immune regulation. Treg do so by utilizing the transcription factor program of the population they are suppressing, or tissue specific transcription factors, respectively (67, 68, 71). For example, Treg that co-express T-BET next to FOXP3 can efficiently suppress Th1 responses (72), whereas the expression of adipose tissue-specific peroxisome proliferator-activated receptor gamma (PPARγ) is needed for Treg to control insulin sensitivity (73). In the latter case, Treg are tissue-resident in a physiological condition and there is an increasing list of tissue specific phenotypes of tissue-resident Treg exerting non-immunological but tissue-homeostatic functions [reviewed in (67)]. To what extend the profile of conventional Trm is applicable to tissue-resident Treg remains to be explored.

**Figure 1:**
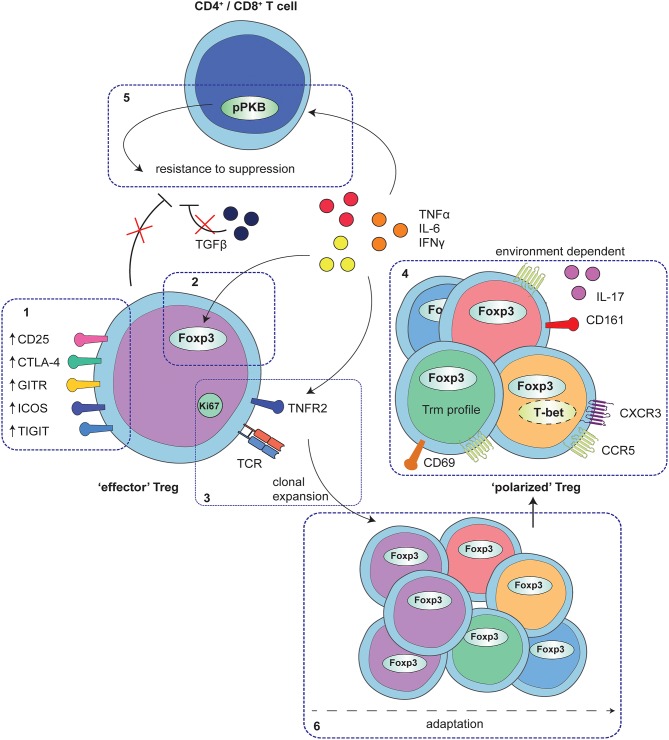
Adaptation of Treg to local auto-immune inflammation. At the site of human autoimmune-inflammation functional Treg are present that display an effector phenotype. This phenotype is characterized by increased expression of functional Treg markers, including CD25, CTLA4, GITR, ICOS, and TIGIT (1) and stable increased expression of Foxp3, and at least in part, instructed by local inflammatory signals (2). Furthermore, Treg are clonally expanding as is reflected by increased Ki67 expression and a local clonal TCR repertoire, possibly mediated by TNFα signaling via TNFR2 on Treg (3). In addition to the effector profile, Treg may also display a specific environment instructed profile, including e.g., expression of CD161 and IL-17 production, upregulation of Th1- and inflammation-associated markers and chemokine receptors and/or characteristics of a Trm profile (4). These polarization profiles are not exclusive but rather are overlapping, depending on the specific local conditions. Whereas, adapted Treg are functional, local cytokines produced by monocytes and fibroblasts also affect CD4^+^ and CD8^+^ T-cells, in part by hyper-phosphorylation of PKB/c-AKT, conferring resistance of local CD4^+^ and CD8^+^ T-cells to Treg suppression (5). Overall, the process of Treg adaptation in inflammatory settings is highly influenced by the local environment, most likely starting with an expanding effector population that can be further fine-tuned with environmental adaptations (6).

Using the proper gating strategy for Treg markers, the frequency of Treg in rheumatic diseases is increased at the site of inflammation in JIA and JDM (74–76). Whether Treg function is impaired in these diseases is still under debate, partly related to differences in phenotyping markers and conditions of *in vitro* assays used to test Treg functionality. Multiple studies have however shown that Treg derived from SF of JIA patients maintain their suppressive function and upregulate Treg functional markers, such as CD25, CTLA4, and GITR, rather pointing toward an eTreg profile (77–79) (Figure 1).

#### Treg Stability

Instability of Treg has long been suspected to play a role in disease pathology. Instability is defined by loss of FOXP3 expression and suppressive function, with a concomitant acquisition of an effector phenotype. The stability of Treg is a contentious issue, with contradicting data from several studies (80). Multiple mouse models, including genetic fate-mapping models that allow tracking Foxp3 expressing cells, revealed that Treg are fairly stable *in vivo* with a small proportion of cells that lose Foxp3 expression (81–84).

At the site of autoimmune inflammation in humans, FOXP3-expressing Treg that produce pro-inflammatory cytokines have been described (85, 86). In specific tissues however, it is unknown if aberrant adapted Treg add to disease pathogenesis. In this regard, it is important to distinguish between functional plasticity/adaptability and lineage instability. In JIA, a small fraction of SF Treg expresses CD161 and is capable of producing pro-inflammatory cytokines. At the same time, FOXP3 expression remains high and suppressive capacity is also maintained (87, 88). Another paper studying Treg stability in SF of JIA patients, demonstrated that the T-cell receptor (TCR) repertoires of Treg is very distinct from conventional T-cells in SF, indicating a different origin and thus excluding a large degree of instability of Treg (89). On top of that, the same paper showed that Treg need inflammatory signals present in SF to maintain their FOXP3 expression, supporting the idea that local signals in an inflammatory environment can stabilize or even enhance the Treg phenotype.

Systemic administration of IL-2 as a therapy to maintain and possibly expand Treg is currently being tested for SLE patients. A recent paper reports on reduced CD25 expression on peripheral blood Treg of SLE patients, that correlates to the reduced production of IL-2 from circulating memory T-cells (90). Since the increased expression of CREMα leads to reduced IL-2 production of effector T-cells, and IL-2 receptor (CD25) signaling via STAT5 is pivotal for maintained Foxp3 expression in Treg, impaired Treg function could be a consequence (91). This provides a rationale for Treg targeted therapy by low dose IL-2 administration (92). However, it is not known whether the reduced CD25 expression on Treg also occurs at the site of inflammation. Moreover, in peripheral blood of active SLE patients enhanced levels of functional, non-cytokine producing Helios^+^ Treg have been identified that positively correlate with disease activity (93, 94). In addition, these cells were shown to express CXCR3 and CCR4, allowing them to migrate to inflammatory sites (93).

The stability of Treg is regulated on multiple levels. The acquisition of a specific epigenetic landscape is a strong determinant (95), as well as transcriptional and post-transcriptional regulation of FOXP3. *In vitro* studies have shown that environmental cues can modulate these processes. For example ubiquitination of FOXP3, that targets its proteosomal degradation, is highly regulated by the ubiquitin ligase Stub1 and the deubiquitinating enzyme USP7. *In vitro*, inflammatory stimuli allow Stub1 to bind FOXP3 and promote its degradation, which is further facilitated by the downregulation of USP7 (96, 97). However, *ex vivo* gene expression analysis of SF Treg from JIA patients shows stable expression of both proteins (Mijnheer et al., unpublished data). This illustrates that *in vivo* regulation of FOXP3 and Treg function is a highly complex organized process in which multiple proteins are involved. Also, depending on the environmental conditions, different proteins can take part in this network, allowing fine-tuning of the cells to a specific environment and further polarization (81). Altogether, there are no indications for Treg instability on a large scale *in vivo*, but inflammation does seem to impact Treg by differentiation toward an eTreg phenotype.

#### Resistance of CD4^+^ and CD8^+^ Cells to Suppression

Unresponsiveness of T-cells to Treg suppression is most likely a normal transient phenomenon during the assembly of an immune response to clear an infectious threat. However, in auto-immune diseases this resistance of local effector T-cells to suppression contributes to a sustained inflammatory response and subsequent disease pathogenesis (98). In JIA, both CD4^+^ and CD8^+^ T-cells from the synovial fluid of affected joints have been found to be intrinsically resistant to suppression (78, 79, 99). The same has been described for CD4^+^ T-cells from SLE patients (100). The resistance to suppression is at least partly mediated by protein kinase B (PKB)/c-akt hyper-activation, induced by local cytokines. TNFα and IL-6 were found to induce resistance in CD4^+^ T-cells, whereas TNFα and autocrine release of IFNγ were responsible for the intrinsic resistance in CD8^+^ T-cells (79, 99). The hyperphosphorylation of PKB/c-akt is interesting in the light of PD-1 signaling, since PD-1 is a strong negative regulator of PKB/c-akt (101). Apparently PD-1 signaling in SF T-cells is not sufficient enough to downregulate this signaling pathway. Blockade of TNFα can restore the susceptibility of CD4 and CD8 T-cells to suppression, which is in line with the effectiveness of therapies targeting TNFα (99, 102). In IBD, resistance of lamina propria T-cells to Treg-mediated suppression has been described as well. In this setting high levels of Smad7, causing insensitivity to TGFβ, have been related to the resistance of effector cells (103). Thus, impaired regulation of the local immune response involves resistance of effector cells, possibly despite functional Tregs.

### Local T-Cell Clonal Expansion

When activated T-cells encounter their cognate antigen in the context of co-stimulation and cytokines, specific clones will expand to initiate a robust adaptive immune response. A diverse repertoire of T-cell receptors (TCRs) of conventional T-cells allows a response to a multitude of possible pathogens. Also thymic derived Treg need a diverse TCR repertoire to regulate auto-immune responses. The TCR repertoire of Treg, representing only a small fraction of the total T-cell pool, is equally diverse as the larger effector pool (104). The need for a diverse (auto-antigen specific) TCR repertoire of Treg has been illustrated by studies using transgenic mice with a restricted TCR repertoire of Treg. In these models, a loss of tolerance toward commensal bacteria and develop autoimmune diseases has been observed (105–108).

At the site of human autoimmune inflammation in both JIA and SLE, clonal T-cell expansions of CD4^+^ T-cell, CD8^+^ T-cell and FOXP3^+^ Treg populations are found (43, 50, 89, 109, 110). Remarkably, especially Treg were found to be hyper-expanded, and to express high levels of Ki67. It is possible that dominant auto-antigens present at the affected sites induce this proliferation (79, 89). Local TNFα can also act as a contributing factor to Treg expansion, as TNFα induces Treg proliferation and effector Treg differentiation via TNFR2 signaling [(111–114); Mijnheer et al., unpublished data]. Since hyper-expanded Treg clones were demonstrated to display a very distinct repertoire compared to conventional CD4^+^ T-cells, local induction of Treg from conventional T-cells is unlikely (89). Tissue resident Treg also show a considerable oligoclonality regarding their TCR repertoire, supporting the notion that tissue Treg and Treg from the site of autoimmune inflammation share typical features (115, 116) (Figure 1). Interestingly, in refractory JIA and JDM patients undergoing hematopoietic stem cell transplantation the TCR repertoire of circulating Treg prior to transplantation was also found to be highly clonal. After transplantation, the Treg TCR repertoire became more diverse over time, except for one patient that experienced a relapse (117). These dates suggest that Treg TCR repertoire abnormalities may contribute to disease pathogenesis, possibly by limiting the chance of antigen encounter, thereby being outcompeted by the less restricted Teff.

Recent data from multiple affected joints in RA show that total TCR repertoires are substantially overlapping (118). In JIA patients, especially PD-1^+^ CD8^+^ T-cells were shown to have a clonal repertoire, with a high overlap in dominant clones between different affected joints (35, 62). The overlap may be explained by the presence of common antigens that drive the disease at multiple sites, and/or (re)circulation of dominant T-cell clones. In affected kidneys of SLE patients expanded CD8^+^ T-cells clones were found to be present for years in sequential biopsies (43), suggesting long-term persistence of dominant T-cell clones. It is tempting to speculate that these dominant T-cell clones play a role in disease relapses, but further studies are need to conclude this.

## Conclusion and Future Perspectives

The expanding field of T-cells, including the discovery of multiple Th subsets as well as observations of mixed phenotypes in inflamed tissues, has made the classification of subsets increasingly complex (5). This complexity, however, likely represents human immunity that is continuously exposed to multiple microorganisms and environmental conditions, in contrast to highly controlled mouse models that have contributed to most of the current knowledge (32, 119). A more nuanced view on T-cells in tissues now arises, with fine-tuning of immune cells to the local environment allowing tailored responses explaining the observed diversity in phenotypes. When inflammation becomes chronic this fine-tuning of T-cells might be unrestrained or insufficient, and as a result cause or contribute to pathogenesis. Potential determinants in this process could be the strength and frequency of TCR stimulation, as well as the presence of absence of CD4 help or co-stimulation, whereas local metabolites also seem to be important. How this is regulated exactly, and what the importance is of different factors remains to be answered, as well as the question what is different in affected human tissue in disease vs. healthy human tissue. The possibilities of gaining new insight are enormous, with newly developed high-throughput technologies that require only small numbers of cells and that allow for analysis of rare T-cell populations from small tissue samples. Single cell sequencing combined with TCR sequencing and mass cytometry on whole tissues will give a deeper understanding about the heterogeneity of T-cells present in human autoimmunity. This, together with smart use of patients samples, such as sequential sampling and sampling from multiple affected sites, will provide novel insights and undoubtedly improve the therapeutic options for patients with rheumatic diseases.

## Author Contributions

GM and FvW have designed the structure and discussed the content of the manuscript. GM has searched the literature and has drafted the text and figure. FvW has critically edited the manuscript.

### Conflict of Interest Statement

The authors declare that the research was conducted in the absence of any commercial or financial relationships that could be construed as a potential conflict of interest.
